# Familial non-medullary thyroid cancer: a critical review

**DOI:** 10.1007/s40618-020-01435-x

**Published:** 2020-10-06

**Authors:** M. Capezzone, E. Robenshtok, S. Cantara, M. G. Castagna

**Affiliations:** 1 Section of Endocrinology and Metabolism, Department of Medical, Surgical and Neurological Sciences, University of Siena, Policlinico Santa Maria alle Scotte, Viale Bracci 1, 53100 Siena, Italy; 2 Institute of Endocrinology, Rabin Medical Center-Beilinson Hospital, Petach Tikva, Israel; 3 Sackler Faculty of Medicine, Tel Aviv University, Tel Aviv, Israel

**Keywords:** Familial papillary thyroid cancer, Familial non medullary thyroid cancer, Syndromic thyroid cancer

## Abstract

**Background:**

Familial non-medullary thyroid carcinoma (FNMTC), mainly of papillary histotype (FPTC), is defined by the presence of the disease in two or more first-degree relatives in the absence of other known familial syndromes. With the increasing incidence of PTC in the recent years, the familial form of the disease has also become more common than previously reported and constitutes nearly 10% of all thyroid cancers. Many aspects of FNMTC are debated, concerning both clinical and genetic aspects. Several studies reported that, in comparison with sporadic PTCs, FPTCs are more aggressive at disease presentation, while other authors reported no differences in the clinical behavior of sporadic and familial PTCs. For this reason, recent guidelines do not recommend screening of family members of patients with diagnosis of differentiated thyroid cancer (DTC). FNMTC is described as a polygenic disorder associated with multiple low- to moderate-penetrance susceptibility genes and incomplete penetrance. At the moment, the genetic factors contributing to the development of FNMTC remain poorly understood, though many putative genes have been proposed in the recent years.

**Purpose:**

Based on current literature and our experience with FNMTC, in this review, we critically discussed the most relevant controversies, including its definition, the genetic background and some clinical aspects as screening and treatment.

## Definition of familial cancer

A comprehensive and accurate family cancer history is essential for cancer risk assessment, reflecting complex interactions among inherited genetic susceptibilities, shared environmental and behavioral factors [1]. An estimated 20% of cancer patients have a positive family history of cancer without the specified criteria for hereditary cancer, and are generally at a moderately increased risk of developing cancer when compared with the general population. These *Familial cancers* (*FC*), defined by the diagnosis of the same type of cancer in two or more first-degree relatives in the absence of known germline mutations, have been described for most major organ systems. A list of the definitions of most frequent familial cancers is shown in Table 1 [2–7].

**Table 1:** Definition of human familial cancers other than carcinoma of the thyroid

Organ	Definition of familial cancer	Ref.
Pancreas	Two or more relatives affected with pancreatic cancer	[2]
Lung	One first or second degree relative with lung cancer	[3]
Gastric	Two first- or second-degree relatives before the age of 50 years or three first- or second-degree relatives independent of age	[4]
Breast–ovarian	(a) Two cases in family members below 50 years or ovarian, peritoneal or tubal cancer in family members at any age; (b) Three cases of breast cancer or ovarian, peritoneal or tubal cancer in family members at any age; (c) One case of breast cancer in a family member below the age of 50 years or bilateral breast cancer in a family member at any age	[5]
Melanoma	Two first-degree relatives or three or more on the same side of the family (irrespective of degree of relationship)	[6]
Prostate	Two first-degree relatives at any age, or one first-degree relative and two or more second-degree relatives diagnosed at any age	[7]

## Epidemiology of familial cancer

Epidemiologists used family history (FH), usually of first-degree relatives (FDRs), as a marker for genetic risk, knowing that FH reflects the consequences of genetic susceptibilities, shared environment, and common behaviors.

Individuals with a positive family history for certain types of cancers can have an increased risk of developing cancer themselves. Several studies indicated that individuals with a family history of cancer are two- to threefold more susceptible to develop the same cancer than those without such a history [8]. To evaluate the rate of familial cancers, population-based studies were used but are limited if a disease or its hereditary form is rare. Case–control study design has also been employed, but again, if most cases of disease are sporadic, there may be insufficient power to detect aggregation within families. As a remedy, other studies recruited subjects on the basis of their family history of disease [9]. Nevertheless, the magnitude of the association with family history varies between studies, cancer sites, and strata of sex and age, being generally stronger for younger probands. The prevalence of a family history of breast cancer has been estimated to range from 5 to 22%, of colon cancer from 2.0 to 9.4%, of ovarian cancer from 1.1 to 3.5%, of endometrial cancer from 0.5 to 1.4%, and of prostate cancer from 4.6 to 9.5% [10].

For these reason, it is important to consider that when family history is used to assess the risk of cancer, its accuracy and completeness must be taken into account. In fact, it may happen that the family history is reported incorrectly or that the person is not properly aware of family members with cancer. Furthermore, sometimes small families or premature deaths may limit the information obtainable from the family history itself.

## Familial non-medullary thyroid carcinoma (FNMTC)

### Controversy in the definition of FNMTC

In 1955, Robinson and Orr published the first report of isolated familial papillary thyroid cancer affecting 24-year-old identical twins [11]. Since that time, many reports have been published about the presence of kindreds with differentiated thyroid cancer (DTC) suggesting the real existence of a familiar form of non-medullary thyroid cancer (FNMTC). The accepted definition of FNMTC is the occurrence of the disease in two or more first-degree relatives of the patient [12]. The traditional definition of FNMTC was based primarily on large population-based analysis, where a national cancer registry was present that documented how the relatives of non-medullary thyroid cancer patients had a higher risk of developing the same type of cancer, often the highest among all cancer types [13, 14]. However, this definition has been discussed since the presence of only two affected members in kindreds may represent a fortuitous association of the disease, as suggested by Charkes [15], who applied an exact probability measure to a series of first-degree family members with FNMTC. According to his mathematical simulation, 62–69% of 2-hit families are sporadic occurrences and thus, only families with 3 or more affected first-degree relatives should be considered for clinical and genetic investigations of FNMTC. However, recently a paper from the Swedish Family Cancer database that included 14.7 million individuals, was used to estimate familial cancer risk for the 25 most common cancer sites and has been highlighted as the first-degree relatives of patients affected by small intestines, thyroid and testicular cancers are those most at risk of developing the same cancer [8]. If a parent was affected, risk for offspring was threefold for thyroid cancer. The same authors observed that more than 90% of familial cancers were in families with two affected members, with the exception of prostate cancer (87%). Families with 3 or more concordant cases accounted for more than 1% of all cancer only for prostate and breast cancer. The authors hypothesized that the low proportion of families with 3 or more affect individuals is likely to define the genetic architecture of familial cancer; high-penetrance predisposition is rare compared to low-penetrance risk predisposition signaled by two-case families. Since ionizing radiation is the only established environmental risk factor for thyroid cancer, the majority of familial cases where two family members were affected, are probably caused by low-penetrance genes [8]. More recent data suggest that FNMTC is a polygenic disorder with variable penetrance likely associated with a low-to-moderate number of low-penetrant alleles [16]. Genome-wide association studies have pinpointed at least ten loci having odds ratios of ~ 1.2 to ~ 1.8, indicating that low-penetrance predisposition variants play important roles. Therefore, the presence of only two affects does not exclude familial tumor. Such features, as the presence of genetic anticipation, should also be considered in the definition of FNMTC. The age of onset of the tumor, even more than the number of affected family members, is an important parameter for the existence and likelihood of a family-type tumor. In fact, familial risks tend to decrease with the age of diagnosis of the probands for some cancer sites [17]. The presence of genetic anticipation has been demonstrated in FNMTC patients suggesting the inheritance of this familial form of thyroid cancer even in cases where only two members of a family are affected [18]. A potential bias when diagnosing FNMTC could be related with the presence of familial multinodular goiter (MNG). In areas with iodine deficiency MNG is common and, although the population incidence of MNG far exceeds that of thyroid cancer, there is also evidence that genetic susceptibility to MNG and to NMTC may be related [19]. In fact, families segregating an autosomal dominant trait for MNG often include cases of NMTC. Several studies have reported genetically linked loci for familial MNG and actually, FNMTC and its relationship with familial MNG are now recognized clinical entities with well identified MNG loci [20].

## Does a driving gene for familial non-medullary thyroid cancer exist?

The presence of a genetic basis for FNMTC is controversial. For familial forms, at least three distinct clinical presentations have been reported: (1) a particularly rare syndromic familial form, in which thyroid carcinoma is associated with non-thyroid diseases such as Gardner syndrome, Cowden syndrome, Werner’s syndrome or Carney complex in which a responsible gene has been identified; (2) a second phenotype, also very rare, in which familial thyroid cancer is associated with a particular histotype with well-defined susceptibility loci; and (3) the most frequent form, where familial papillary thyroid cancer (FPTC) is the only clinical manifestation in the absence of candidate genes. The syndromic group is characterized by a preponderance of non-thyroidal tumors and is associated with Mendelian cancer syndromes. The associated syndromes include Cowden syndrome, familial adenomatous polyposis (FAP) with its form Gardner syndrome, Carney complex, Werner and DICER1 syndromes [21–26] [Table 2].

**Table 2:** Syndromes associated with familial non-medullary thyroid cancer (FNMTC)

Syndrome	GENE	Types of thyroid cancer (incidence %)	Ref.
Familial adenomatous polyposis	APC	Cribriform papillary thyroid cancer (2–12%)	[22]
Cowden syndrome	PTEN	Classical or follicular papillary thyroid cancer (> 10%)	[23]
Carney complex type 1	PRKARI	Follicular or Papillary thyroid cancer (4–60%)	[24]
Werner syndrome	WRN	Follicular, Papillary or Anaplastic thyroid cancer (18%)	[25]
DICER 1 syndrome	DICER1	Multinodular thyroid hyperplasia and carcinoma ()	[26]

To give reason for all non-syndromic FNMTC cases, some genomic regions considered as FNMTC predisposing loci have been reported in various studies. The first “locus” (called “thyroid carcinoma with oxyphilia”, TCO) was located at position 19p13.2 in a single French family with a rare form of familial oxyphilic cell thyroid tumor (OMIM# 603386) [27]. The TCO linkage was subsequently confirmed in other pedigrees although the phenotype of cell oxyphilia was associated only in few cases [28]. Another locus has been identified at position 1q21 in an American family affected by both FNMTC and papillary neoplasia of the kidney (“papillary thyroid carcinoma and papillary renal neoplasia”, fPTC/PRN) [29]. A further susceptibility locus (“non-medullary thyroid carcinoma 1″, NMTC1) was identified on chromosome 2q21 in a large family from Tasmania suffering from a high frequency of papillary carcinoma follicular variant [30]. Association between TCO and NMTC loci has also been reported [31] with the consequence of an increased risk in individuals that inherit both susceptibility genes. Loci associated with FNMTC are reported in Table 3.

**Table 3:** Susceptibility loci associated with familial non-medullary thyroid cancer (FNMTC)

Tumor	Chromosomal loci	Numero OMIM	Ref.
TCO	19q13.2	606240	[27]
PTC/PRN	1q21	606240	[29]
NMTC1	2q21	6063831	[30]

*PTC* Papillary thyroid cancer, *PRN* papillary renal neoplasm, *TCO* Thyroid tumors with cell oxyphilia

For isolated FNMTC, no susceptibility locus was found and no germline mutations were demonstrated. The classic genetic alterations most commonly described in somatic PTC (point mutations of BRAF and RAS and rearrangements of RET/PTC, PPARɣ and TRK) have not been demonstrated at germline level in FNMTC. The hypothesis that FNMTC is a hereditable cancer, has been suggested by epidemiological studies and by the analysis of some national cancer registries which have shown that, among all types of cancer, non-medullary thyroid carcinoma shows a significantly higher risk of developing in the first-degree relatives of an affected member compared to the general population [13, 14]. The observation that the first degree relatives of an affected individual have an eight- to tenfold increased risk of developing the disease suggest that there must be an underlying germline mutation(s) that have not been discovered, yet. The presence of family forms for NMTC is described as a polygenic disorder associated with multiple genes with low to moderate or incomplete penetrance and variable expression as observed on reports of families with three or more affected members, horizontal transmission in siblings and increased percentage of male patients with FNMTC compared to those with sporadic NMTC. Many studies have apparently shown good evidence for putative susceptibility genes, but subsequent analysis has often contradicted these findings. Probably, the disparity in results may be explained by the variation in study designs as the differences in the inclusion criteria. Some studies analyzed FNMTC families with only two affected members whereas others enrolled FNMTC families with at least three first degree relatives to reduce the possibility of a random association of sporadic tumors. In addition, many studies were conducted only on single kindred or in small size families with a variety of additional benign common thyroid disorders. Hypothesized susceptibility genes are reported in Table 4 [32–36]. Among them, there is some evidence that HABP2 G534E variant (chromosome 10q25.3) is a susceptibility gene for FNMTC. In 2015, Gara et al. published in New England J of Medicine [32], the presence of a germline p.G534E variant in the *HABP2* gene in seven affected members of a huge kindred with familial non-medullary thyroid cancer and in 4.7% of 423 patients with sporadic thyroid cancer. *HABP2* gene encodes a member of the peptidase S1 family of serine proteases which bounds hyaluronic acid playing a role in the coagulation and fibrinolysis systems. In the study of Gara et al., the variant was associated with increased HABP2 protein expression in tumor samples from affected family members, as compared with normal adjacent thyroid tissue and sporadic cancers. Functional studies showed that HABP2 had a tumor-suppressive effect, whereas the G534E variant results in loss of function. These observations lead the authors to conclude that the *HABP2* p.G534E is a susceptibility gene for familial non-medullary thyroid cancer. The impact of these results prompted several authors to search for the HABP2 p.G534E variant in other kindreds. All these studies failed in detecting the p.G534E mutation in FNMTC suggesting that Gara et al. identified a private variation [37, 38]. However, recently, a study by Colombo et al., revealed that the HABP2 p.G534E does not segregate with cancer but its mRNA had a very variable expression in tissues from FNMTC, sporadic papillary thyroid cancers (PTCs) or contralateral normal tissues opening again a question on *HABP2* role in thyroid cancer [39]. Some studies have demonstrated that FNMTC patients have shorter germline and somatic telomere length suggesting the role of telomere shortening in the development of FNMTC [40–42]. A recent paper reinforced these data, showing that the percentage of patients with short RTL was greater among those with FPTC than among those with sporadic PTCs and showed that leukocyte telomere length was significantly associated with PTC risk. These findings supported the hypothesis that telomere length is significantly correlated with susceptibility to PTC [43]. However, another study did not shown any differences in telomere length and telomerase activity between familial and sporadic PTC cases [44]. The presence of shorter telomeres has been clinically associated with the presence of genetic anticipation with the second generation acquiring the disease at an earlier age and having more advanced disease at presentation, reinforcing the hypothesis that FNMTC is a true familial disease rather than the fortuitous association of the same disease in a family [18]. In conclusion, at the moment, a known driving gene for familial non-medullary thyroid cancer has not been identified. Many published pedigrees suggest an autosomal dominant mode of inheritance with reduced penetrance, but polygenic inheritance cannot be excluded. For this reason, it would be appropriate to create, in agreement with all the medical centers that deal with this type of research, an international consortium for the recruitment of families with recurrence of NMTC and to allow the creation of a wide database and tissue bank where to collect a large quantity of material. This possibility would allow to carry out large-scale studies, to standardize FNMTC patient selection criteria and the application of the new molecular research techniques in an attempt to discover the pathogenetic mechanism(s) that leads to FNMTC development.

**Table 4:** Susceptibility genes associated with familial non-medullary thyroid cancer (FNMTC)

References	Location	Gene
[32]	10q25.3	HABP2
[33]	12q14	SRGAP1
[34]	9pq22.33	FOXE1
[35]	14q13	TITF-1/NKX2.1
[36]	15q23	MAP2K5

## Controversies in management of FNMTC

Families with FNMTC present a unique challenge in clinical practice, as question regarding the need for screening and the need for aggressive initial therapy remain controversial [45, 46]. While the debate regarding these topics has been going on for years, the context of these questions has changed with our current understanding of sporadic disease, including recognition of the large reservoir of DTC in the general population, the risks associated with over-diagnosis and over-treatment, and the trend for less aggressive therapy for early-stage DTC. We, therefore, address the topics of screening and extent of therapy in light of current literature.

### Screening

While screening for non-familial DTC is considered unnecessary or even potentially harmful [47], some authors have proposed annual screening in families with FNMTC. Advocates of screening point to higher rates of DTC in first-degree kindreds, and to several studies which demonstrated more advanced disease at presentation leading to worse outcome in these families, though data on these topics are conflicting.

A recent prospective study with yearly screening of at-risk individuals from families with FNMTC at the National Institutes of Health (NIH) by Klubo-Gwiezdzinska et al. [48] found thyroid cancer by ultrasound screening in 4.6% individuals from families with two members affected (similar to the estimated prevalence in the general population of 4.5%) [16, 49] and in 22.7% of members from families with three or more patients affected. Similar results were reported in a prospective study by Rios et al. [50], which screened families with mostly two affected members, and found 5.5% incidence of thyroid cancer. Using a mathematical analysis of the SEER (Surveillance Epidemiology and End Results) database, Charkes [15] concluded that up to 62% of 2-hit families may be sporadic occurrences, while the chance of sporadic DTC in families of three or more affected members is less than 6%. These data highlight the need to differentiate between screening in the relatively common 2-hit families with relatively low DTC detection rate, from those with three or more affected members where high detection rate is expected from first degree screening.

Screening would also be recommended if early detection and treatment would lead to decreased mortality or improved quality of life in affected families. Unfortunately, no interventional screening programs have reported these outcomes. Currently, proponents of screening point to some (but not all) studies reporting of disease appearance at an earlier age [16, 45, 51–55], and studies reporting more advanced disease at presentation leading to worse outcomes, especially in patients with three or more affected members [45, 53, 54, 56–60]. However, published studies are conflicting, with one meta-analysis and several studies reporting clinical course and outcome similar to sporadic disease [61–64].

Current guidelines, including the adult and pediatric ATA guidelines [65, 66], do not recommend for or against U/S screening in non-syndromic FNMTC, apart from routine physical examination. However, in light of the recent NIH study discussed above, it seems reasonable to recommend screening with yearly U/S in kindred with three or more affected family members, starting from the age of 20 or 10 years before the earliest age of the diagnosis in the family. For families with only two affected family members, there are insufficient data to recommend yearly U/S screening, given the likelihood of sporadic disease aggregated in one family, and the risk of over-diagnosis and over-treatment. In cases of syndromic familial DTC, published recommendation are available for screening in PTEN hamartoma tumor syndrome and APC-associated polyposis [67–69].

### Extent of therapy

To determine whether patients with FNMTC require more aggressive therapy, it is important to differentiate between studies evaluating disease presentation (comparing consecutive patients with FNMTC with consecutive patients with sporadic disease, often with different baseline characteristics) [16, 18, 51–54, 56, 57, 60, 64, 70–73], from studies evaluating outcome in groups which are matched for baseline characteristics [61, 63, 74].

While a more aggressive disease at presentation (larger tumors, more extra-thyroidal extension or more lymph nodes involvement) would serve as an argument for screening, it does not imply that standard risk-adjusted therapy would not be effective for these patients. For example, if on average patients with FNMTC have more lymph node involvement at presentation (and therefore more recurrences), it does not imply that a patient with a familial 1.1-cm intra-thyroidal PTC would benefit from higher activities of radioiodine therapy as compared to a similar patient with sporadic disease. Hence, the extent of therapy should be guided by controlled studies with comparable groups, with a focus on response to therapy and disease outcome.

In a multicenter case–control study by Alsanea et al. published in 2000, 48 American and Japanese patients with FNMTC were matched with 114 patients with sporadic disease for age, gender, stage at presentation, and history of radiation exposure [74]. Patients with FNMTC had a significantly shorter disease-free survival, with the worst outcome in patients with distant metastases and with more than two affected family members. In contrast, Cao et al. [61] published a large study in 2016 of 372 Chinese patients with FNMTC matched with 372 sporadic cases for gender, age, tumor/node/metastasis (TNM) staging, and approximate duration of follow-up. There was no significant difference in recurrences (7.8% vs. 5.4%, respectively) or disease-related mortality (one patient in each group), with a significant difference only in the < 1 cm subgroup recurrence rate (7.3 vs. 1.3%, *p *= 0.002). Similar outcomes were also demonstrated in a small Canadian matched case–control study from 2004 [63] where 24 FNMTC cases were matched with 24 sporadic cases for age, gender, stage, and tumor size. There was no difference in surgical management or recurrence between the two groups.

Multiple other studies compared consecutive patients with FNMTC with sporadic cases (often with different baseline characteristics), with mixed results in terms of disease outcome. Most studies reported of more aggressive disease at presentation leading to worse outcome in the FNMTC group [18, 52–54, 70]. However, several studies reported of more aggressive disease at presentation with similar outcome at the end of follow-up [60, 64, 71, 73, 75], or of similar baseline characteristics with similar outcome [18, 72] or worse outcome [57]. These differences may result from the retrospective design of the studies, small sample size, inclusion of families with both two and three affected kindreds, and genetic variability across populations.

Overall, patients with FNMTC tend to have more advanced disease at presentation and, therefore, tend to receive more aggressive initial therapy. However, there are no data to suggest their response to initial therapy (surgery or radioiodine) is different compared to sporadic cases, and therefore, there is insufficient evidence to suggest more aggressive surgery or higher activities of radioiodine, above what would be usually recommended for a specific disease stage. It is worth noting, however, that current trends of less aggressive therapy for sporadic DTC were not evaluated in families with FNMTC, and treatment with total thyroidectomy and radioiodine is reasonable, especially in patients with three or more affected members.

## Conclusions

Familial non-medullary thyroid carcinoma represents an interesting challenge for clinician because many aspects of this disease still need to be clarified, from its definition to genetic and clinical aspects. There are conflicting data on aggressiveness of FNMTC compared to sporadic form and the latest ATA guidelines do not recommend a different therapeutic approach for FNMTC patients. However, the possibility of a family history as a risk factor for DTC underlines the importance of an accurate anamnesis. Some clinical parameters such as the diagnosis of FNMTC at a relative young age associated with the presence of tumor multicentricity and/or bilaterality are elements that must draw attention to a possible form of familial cancer. The availability of a specific genetic marker in the future will allow a diagnosis of certainty and individually tailor the optimal screening and treatment.
